# Crystal structure of 2,2′-bi­pyrrole

**DOI:** 10.1107/S2056989017013433

**Published:** 2017-09-25

**Authors:** Christopher A. Michaels, Lev N. Zakharov, Ashley N. Lamm

**Affiliations:** a Department of Chemistry and Biochemistry, Eastern Washington University, Science 226, Cheney, WA 99004, USA; b Department of Chemistry, 1253 University of Oregon, Eugene, Oregon 97403, USA

**Keywords:** crystal structure, bi­pyrrole, N—H⋯π inter­actions, herringbone structure

## Abstract

The complete mol­ecule of the title compound, C_8_H_8_N_2_, is generated by a crystallographic center of symmetry. In the crystal, short N—H⋯π [H⋯π = 2.499 (19) Å] inter­actions link the mol­ecules into a herringbone structure.

## Abstract 2

The crystal structure of the centrosymmetric title compound features short N—H⋯π inter­actions.

## Chemical context   

Bi­pyrrole, C_8_H_8_N_2_, has been studied extensively over the years: the first reported synthesis was in 1962 (Rapoport & Castagnoli, 1962 ▸). The bi­pyrrole core occurs in naturally occurring compounds such as prodigiosin (Wasserman *et al.*, 1960 ▸). Functionalized bi­pyrroles have been shown to have anti-cancer activity (Manderville, 2001 ▸). Corrole rings contain a bi­pyrrole segment in the macrocycle (Aviv-Harel & Gross, 2009 ▸). Herein, we report on the crystal structure of the title compound, (I), synthesized by the oxidative coupling of pyrrole.
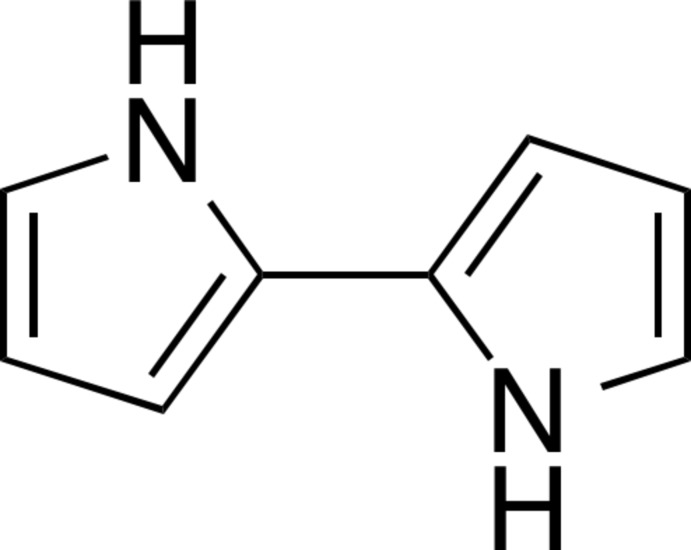


## Structural commentary   

**Figure 1:**
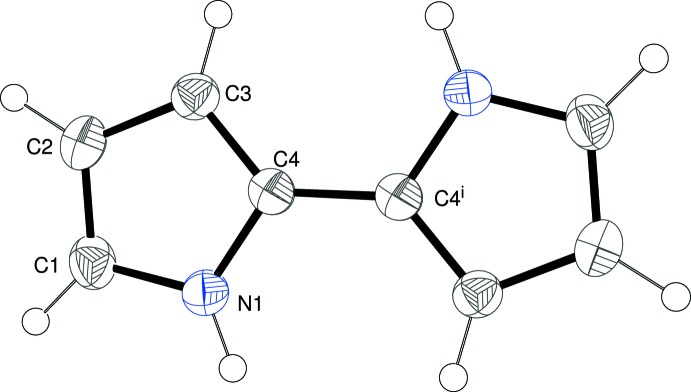
The mol­ecular structure of the title compound with displacement ellipsoids drawn at the 50% probability level. [Symmetry code: (i) 1 − *x*, 2 − *y*, −*z*.]

The complete mol­ecule of (I) is generated by a crystallographic center of symmetry (Fig. 1 ▸), and therefore the pyrrole rings are exactly parallel and the N—H groups face in opposite directions. The C2—C3 bond in (I) is 1.4151 (19) Å, *versus* the equivalent bond in pyrrole, which has a length of 1.423 (3) Å. The double bonds C1=C2 and C3=C4 in (I) are 1.3635 (19) and 1.3767 (17) Å, respectively, *versus* the double-bond length in pyrrole of 1.357 Å. The shortening of the C2—C3 bond and the lengthening of the adjacent C=C double bonds in (I) compared to pyrrole is consistent with stronger inter­molecular inter­actions (see below).

## Supra­molecular features   

**Figure 2:**
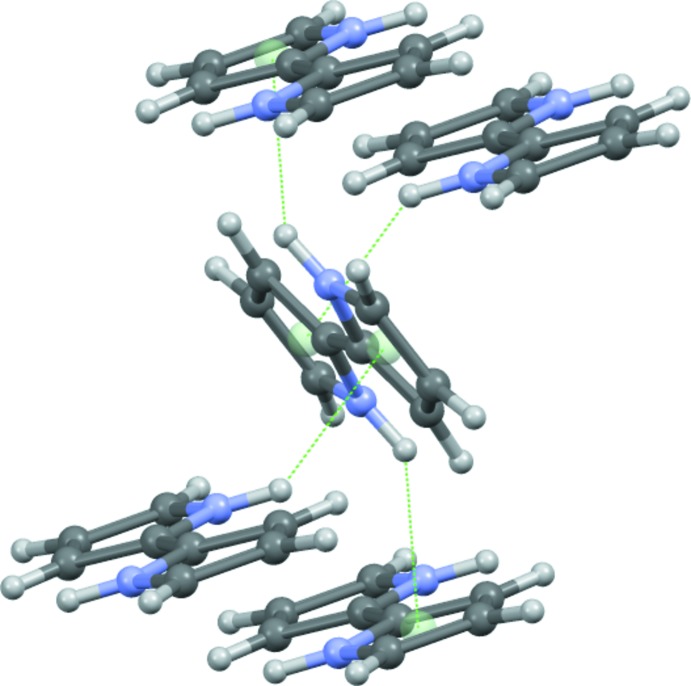
View of the N—H⋯π inter­actions and packing in the title compound.

In the crystal of (I), the mol­ecules adopt an edge-to-face orientation: the distance between the N—H group and the centroid of the adjacent pyrrole ring generated by the 2_1_ screw axis is 2.499 (19) Å (Table 1 ▸). A survey of the Cambridge Crystallographic Database (Groom *et al.*, 2016 ▸) showed that the average N—H⋯π separation is 2.804 Å and 13 out of 156 inter­actions (8.3%) had an N—H⋯π inter­actions shorter than 2.5 Å. The crystal packing in (I) is similar to that of pyrrole, which has the same inter­molecular N—H⋯π inter­actions (Goddard *et al.*, 1997 ▸). The edge-to-face inter­action has been suggested to be a stabilizing factor in protein structures and polypeptides have been observed to have a separation of 2.42 Å from the N—H group to the phenyl ring (Steiner, 1998 ▸) and calculations corroborate these data (Levitt Perutz, 1988 ▸). It is notable that the N—H bond of 2,2′-bi­pyrrole points almost directly at the midpoint of the C2—C3 bond. In the extended structure, the mol­ecules are orientated by edge-to-face inter­actions generating a herringbone pattern, see Fig. 2 ▸.

## Database survey   

There are very few examples of similar compounds available in the literature. Most bi­pyrroles have carbonyl groups as substituents on the pyrrole ring in which the N—H group forms hydrogen bonds with the oxygen atom of the carbonyl (Okawara *et al.*, 2015 ▸). So far as we are aware, there are no bi­pyrrole examples that exhibit the same packing and hydrogen-bonding pattern as the title compound; the closest example is pyrrole itself (Goddard *et al.*, 1997 ▸).

## Synthesis and crystallization   

230 µl (3.3 mmol) of pyrrole was added to di­chloro­methane (10 ml), degassed and cooled to 195 K. Tri­methyl­silyl bromide (290 µl, 2.2 mmol) and phenyl iodine trifluoracetic acid (477 mg, 1.1 mmol in 1 ml di­chloro­methane) were added quickly to the cooled reaction. The mixture was stirred for 1 h then extracted with a saturated sodium bicarbonate solution and purified by column chromatography with a penta­ne/ethyl acetate (1:1) solution. Colourless blocks of (I) were obtained 7 d later by slow evaporation from an ethyl acetate solution.

## Refinement   

Crystal data, data collection and structure refinement details are summarized in Table 2 ▸. The H atoms were located in difference maps and refined with isotropic displacement parameters.

## Supplementary Material

Crystal structure: contains datablock(s) I. DOI: 10.1107/S2056989017013433/hb7692sup1.cif

Structure factors: contains datablock(s) I. DOI: 10.1107/S2056989017013433/hb7692Isup2.hkl

Survey of the Cambridge Database NH-pi bond lengths. DOI: 10.1107/S2056989017013433/hb7692sup3.pdf

CCDC reference: 1575394

Additional supporting information:  crystallographic information; 3D view; checkCIF report

## Figures and Tables

**Table 1:** Hydrogen-bond geometry (Å, °) *Cg* is the centroid of the N1/C1–C4 ring.

*D*—H⋯*A*	*D*—H	H⋯*A*	*D*⋯*A*	*D*—H⋯*A*
N1—H1*N*⋯*Cg*1^i^	0.907 (16)	2.499 (19)	3.2275 (12)	138.1 (13)

Symmetry code: (i) 
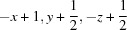
.

**Table 2:** Experimental details

Crystal data	
Chemical formula	C_8_H_8_N_2_
*M*_r_	132.16
Crystal system, space group	Monoclinic, *P*2_1_/*c*
Temperature (K)	173
*a*, *b*, *c* (Å)	5.9500 (2), 6.7650 (2), 8.4363 (3)
β (°)	96.746 (2)
*V* (Å^3^)	337.22 (2)
*Z*	2
Radiation type	Cu *K*α
μ (mm^−1^)	0.64
Crystal size (mm)	0.11 × 0.10 × 0.06

Data collection	
Diffractometer	Bruker APEXII CCD
Absorption correction	Multi-scan (*SADABS*; Bruker, 2004 ▸)
*T*_min_, *T*_max_	0.666, 0.753
No. of measured, independent and observed [*I* > 2σ(*I*)] reflections	1989, 590, 540
*R*_int_	0.026
(sin θ/λ)_max_ (Å^−1^)	0.595

Refinement	
*R*[*F* ^2^ > 2σ(*F* ^2^)], *wR*(*F* ^2^), *S*	0.034, 0.098, 1.08
No. of reflections	590
No. of parameters	62
H-atom treatment	All H-atom parameters refined
Δρ_max_, Δρ_min_ (e Å^−3^)	0.18, −0.16

Computer programs: *APEX2* and *SAINT* (Bruker, 2004 ▸), *SHELXS97* and *SHELXTL* (Sheldrick, 2008 ▸) and *SHELXL2014* (Sheldrick, 2015 ▸).

## supplementary crystallographic information

### Crystal data

**Table:**

C_8_H_8_N_2_	*F*(000) = 140
*M**_r_* = 132.16	*D*_x_ = 1.302 Mg m^−^^3^
Monoclinic, *P*2_1_/*c*	Cu *K*α radiation, λ = 1.54178 Å
*a* = 5.9500 (2) Å	Cell parameters from 1086 reflections
*b* = 6.7650 (2) Å	θ = 7.5–66.5°
*c* = 8.4363 (3) Å	µ = 0.64 mm^−^^1^
β = 96.746 (2)°	*T* = 173 K
*V* = 337.22 (2) Å^3^	Cut-block, colorless
*Z* = 2	0.11 × 0.10 × 0.06 mm

### Data collection

**Table:**

Bruker APEXII CCD diffractometer	540 reflections with *I* > 2σ(*I*)
Radiation source: Incoatec IµS	*R*_int_ = 0.026
φ and ω scans	θ_max_ = 66.5°, θ_min_ = 7.5°
Absorption correction: multi-scan (SADABS; Bruker, 2004)	*h* = −7→4
*T*_min_ = 0.666, *T*_max_ = 0.753	*k* = −7→8
1989 measured reflections	*l* = −9→10
590 independent reflections	

### Refinement

**Table:**

Refinement on *F*^2^	Primary atom site location: structure-invariant direct methods
Least-squares matrix: full	Hydrogen site location: difference Fourier map
*R*[*F*^2^ > 2σ(*F*^2^)] = 0.034	All H-atom parameters refined
*wR*(*F*^2^) = 0.098	*w* = 1/[σ^2^(*F*_o_^2^) + (0.0657*P*)^2^ + 0.034*P*] where *P* = (*F*_o_^2^ + 2*F*_c_^2^)/3
*S* = 1.08	(Δ/σ)_max_ < 0.001
590 reflections	Δρ_max_ = 0.18 e Å^−^^3^
62 parameters	Δρ_min_ = −0.16 e Å^−^^3^
0 restraints	

### Special details

**Table:**

Geometry. All esds (except the esd in the dihedral angle between two l.s. planes) are estimated using the full covariance matrix. The cell esds are taken into account individually in the estimation of esds in distances, angles and torsion angles; correlations between esds in cell parameters are only used when they are defined by crystal symmetry. An approximate (isotropic) treatment of cell esds is used for estimating esds involving l.s. planes.

### Fractional atomic coordinates and isotropic or equivalent isotropic displacement parameters (Å^2^)

**Table:**

	*x*	*y*	*z*	*U*_iso_*/*U*_eq_	
N1	0.53346 (18)	1.09945 (15)	0.20670 (12)	0.0322 (4)	
C1	0.7071 (2)	1.07425 (18)	0.32546 (15)	0.0352 (4)	
C2	0.8665 (2)	0.95786 (18)	0.26832 (16)	0.0330 (4)	
C3	0.7857 (2)	0.91019 (17)	0.10841 (15)	0.0297 (4)	
C4	0.57828 (19)	0.99966 (16)	0.07196 (13)	0.0263 (4)	
H1	0.704 (2)	1.135 (2)	0.4292 (17)	0.043 (4)*	
H1N	0.407 (2)	1.170 (3)	0.2168 (17)	0.046 (4)*	
H2	1.010 (3)	0.916 (2)	0.3269 (19)	0.041 (4)*	
H3	0.859 (2)	0.831 (2)	0.0354 (16)	0.039 (4)*	

### Atomic displacement parameters (Å^2^)

**Table:**

	*U*^11^	*U*^22^	*U*^33^	*U*^12^	*U*^13^	*U*^23^
N1	0.0364 (6)	0.0295 (6)	0.0299 (6)	0.0075 (4)	0.0010 (5)	−0.0030 (4)
C1	0.0424 (8)	0.0320 (7)	0.0296 (7)	0.0003 (5)	−0.0028 (6)	−0.0038 (5)
C2	0.0299 (7)	0.0329 (7)	0.0347 (7)	−0.0016 (5)	−0.0023 (5)	0.0043 (5)
C3	0.0278 (7)	0.0307 (7)	0.0312 (7)	0.0005 (5)	0.0055 (5)	0.0021 (5)
C4	0.0306 (6)	0.0217 (6)	0.0270 (6)	−0.0017 (4)	0.0055 (5)	0.0025 (4)

### Geometric parameters (Å, º)

**Table:**

N1—C1	1.3628 (18)	C2—C3	1.4151 (19)
N1—C4	1.3748 (15)	C2—H2	0.979 (16)
N1—H1N	0.907 (16)	C3—C4	1.3767 (17)
C1—C2	1.3635 (19)	C3—H3	0.959 (14)
C1—H1	0.970 (15)	C4—C4^i^	1.441 (2)
			
C1—N1—C4	110.02 (11)	C3—C2—H2	126.4 (9)
C1—N1—H1N	124.3 (9)	C4—C3—C2	107.93 (11)
C4—N1—H1N	125.6 (9)	C4—C3—H3	124.4 (8)
N1—C1—C2	108.12 (11)	C2—C3—H3	127.6 (8)
N1—C1—H1	121.2 (9)	N1—C4—C3	106.69 (11)
C2—C1—H1	130.7 (9)	N1—C4—C4^i^	121.74 (13)
C1—C2—C3	107.23 (11)	C3—C4—C4^i^	131.57 (13)
C1—C2—H2	126.4 (9)		
			
C4—N1—C1—C2	0.11 (14)	C1—N1—C4—C4^i^	179.68 (12)
N1—C1—C2—C3	−0.15 (14)	C2—C3—C4—N1	−0.07 (13)
C1—C2—C3—C4	0.13 (14)	C2—C3—C4—C4^i^	−179.73 (15)
C1—N1—C4—C3	−0.03 (13)		

Symmetry code: (i) −*x*+1, −*y*+2, −*z*.

### Hydrogen-bond geometry (Å, º)

Cg is the centroid of the N1/C1–C4 ring.

**Table:**

*D*—H···*A*	*D*—H	H···*A*	*D*···*A*	*D*—H···*A*
N1—H1*N*···*Cg*1^ii^	0.907 (16)	2.499 (19)	3.2275 (12)	138.1 (13)

Symmetry code: (ii) −*x*+1, *y*+1/2, −*z*+1/2.
