# Supplementary material for: Crystal structure of 2,2′-bi­pyrrole
Source: Acta Crystallogr E Crystallogr Commun. 2017 Sep 25;73(Pt 10):1517–9. doi: 10.1107/S2056989017013433 (PMC5730307; doi:10.1107/S2056989017013433)
Supplement: Supplementary file 3 [file e-73-01517-sup3.pdf]

	Compound	NH-pi distance
1	AJArim	2.489
2	AJArim	2.959
3	AJISOC	2.683
4	AWIXOT	3.270
5	AWIXOT01	3.349
6	BOHGEM	2.553
7	BOHGOW	3.166
8	CIGKAF	2.863
9	CIGKEJ	2.811
10	CIGKIN	2.600
11	COSKAX	3.033
12	COSKAX	2.472
13	DACPUU	2.456
14	DACPUU	2.678
15	DATZOO	2.905
16	DATZOO	2.883
17	DETLIY	2.663
18	DOJZUY	3.278
19	DUNPUY	2.591
20	DUNPUY	2.61
21	DUNPUY	2.523
22	DUXHAG	3.113
23	DUXHAG	3.086
24	EFACAQ	2.750
25	EPITON	2.630
26	EYAHER	2.992
27	EYAHER	2.845
28	FETPIF	2.521
29	FETPUR	2.920
30	FETPUR	2.598
31	FETQAY	2.662
32	FETQAY	2.72
33	GOLSAB	2.805
34	GOLSAB	2.739
35	GOLSAB01	2.736
36	GOLSAB01	2.731
37	GOQSAI	2.604
38	GOQSAI	2.434
39	GUDJUK	2.685

40	HIJSAW	2.904
41	HIWMUX	2.668
42	HIWMUX	2.648
43	HIWNAE	2.716
44	HIXGAW	3.212
45	HUJTAJ	3.065
46	IFAQAH	2.941
47	IFAQAH	2.586
48	JADHOM	2.768
49	JADHOM	2.72
50	JADHOM	2.628
51	JADHUS	3.038
52	JUBREF	2.751
53	KEQKIC	3.203
54	KEQKOI	2.743
55	KEQKOI	2.815
56	KEQKOI	2.689
57	KEQKUO	2.579
58	KEQKUO	2.603
59	KEQKUO	2.904
60	KISWAM	2.632
61	KISWAM	2.651
62	KUSWIG	2.826
63	KUSWIG	2.892
64	LAFYIA	2.377
65	LAFYIA	2.808
66	LAGBUQ	2.597
67	MIYFUV	2.714
68	MUQHAJ	2.568
69	NAGKIR	2.485
70	NARBUD	2.424
71	NEWKIL	2.989
72	NEWKIL	2.737
73	NOBYEJ	2.616
74	NUGLUY	2.655
75	OBESOE	2.785
76	ORALEZ	2.613
77	ORALEZ	2.698
78	PAHWOM	3.052
79	PILCOE	2.977
80	PILCOE	2.878
81	PILDAR	3.230
82	PILFIB	3.035

83	POXDIP	2.605
84	QABRUI	3.376
85	QANLOG	2.817
86	QANLUM	3.030
87	QANMAT	2.684
88	QIHPOO	3.065
89	QIHPUU	3.076
90	QOMJOT	2.988
91	QUQMAR	2.929
92	QUQMAR	3.185
93	RASTEL	2.500
94	RASTEL01	2.532
95	RASTIP	2.543
96	RASTOV	2.377
97	RASTUB	2.787
98	REHVUV	2.723
99	REHVUV	2.573
100	RENXEP	2.910
101	RENXIT	2.923
102	RENXIT	3.134
103	REVYAT	3.055
104	REYJUB	2.719
105	RIHDIV	2.385
106	RIHDIV	2.521
107	SEBRIC	2.636
108	SEBRIC	2.538
109	TETZOJ	2.641
110	tetzup	2.861
111	TICLUM	2.429
112	TIXYUV	2.409
113	VUTRUY	2.576
114	WEKHAW	2.367
115	WEKHAW	2.87
116	WEPXUL	2.516
117	WEPXUL	2.795
118	WUJCAF	2.496
119	XAMJEC	2.956
120	XAMJEC01	2.999
121	XIBKOI	2.776
122	YOJBAB	3.145
123	YOJBAB	3.103
124	ZURYIW	3.015
125	ALOGAJ	2.434

126	BOHGOW	3.100
127	CATHUC	3.066
128	CIGKAF01	2.892
129	CIGKAF02	3.016
130	CIGKOT	2.958
131	cojpuo	2.803
132	COJQAV	2.663
133	CUZXUS	2.861
134	DEFMIL	3.675
135	DEGBAS	2.552
136	DOSBOD	3.294
137	HUJSUC	2.802
138	HUJTAJ	3.342
139	HUYFIS	2.528
140		2.521
141	JADHUS	2.956
142	LAGBUQ	2.566
143	LUNYEA	2.932
144	NAGKEN	3.243
145	NUGMAF	2.662
146	PAHWOM	3.108
147	PILDAR	2.879
148	PILFIB	3.099
149	RIVLAL	2.722
150	RUXMAA	3.289
151	TAGKET	2.571
152	TICLUM	2.661
153	TIXYUV	2.805
154	VEMTEO	2.594
155	WUJBOS	3.216
156	XAMJEC01	3.168

			ERROR	STDEV
ROMATIC DISTANCE:	2.804	13	0.02029854	0.25352866
